# Expression of Nik-related kinase in smooth muscle cells attenuates vascular inflammation and intimal hyperplasia

**DOI:** 10.18632/aging.103104

**Published:** 2020-04-24

**Authors:** Yi-Jhu Lu, Yee-Jee Jan, Bor-Sheng Ko, Shu-Man Liang, Lujen Chen, Chih-Cheng Wu, Chih-Hui Chin, Cheng-Chin Kuo, Shaw-Fang Yet, Jun-Yang Liou

**Affiliations:** 1 Institute of Cellular and System Medicine, National Health Research Institutes, Zhunan 350, Taiwan; 2 Department of Pathology and Laboratory Medicine, Taichung Veterans General Hospital, Taichung 407, Taiwan; 3 Department of Internal Medicine, National Taiwan University Hospital, Taipei 100, Taiwan; 4 Department of Hematological Oncology, National Taiwan University Cancer Center, Taipei 100, Taiwan; 5 Cardiovascular Center, National Taiwan University Hospital, Hsin-Chu Branch, Hsinchu City 300, Taiwan; 6 Department of Medicine, College of Medicine, National Taiwan University, Taipei 100, Taiwan; 7 Cardiovascular Center, Cathay General Hospital, Taipei 106, Taiwan; 8 Graduate Institute of Biomedical Sciences, China Medical University, Taichung 404, Taiwan

**Keywords:** inflammation, intimal hyperplasia, Nik-related kinase, resveratrol, smooth muscle cell

## Abstract

Inflammation of the vascular microenvironment modulates distinct types of vascular cells, and plays important roles in promoting atherosclerosis, stenosis/restenosis, and vascular-related diseases. Nik-related kinase (Nrk), a member of the Ste20-type kinase family, has been reported to be selectively expressed in embryonic skeletal muscle. However, whether Nrk is expressed in adult vascular smooth muscle, and if it influences intimal hyperplasia is unclear. Here, we found that Nrk is abundantly expressed in cultured vascular smooth muscle cells (VSMC) and mouse arterial intima. Treatment of mouse VSMCs with lipopolysaccharide (LPS) or platelet-derived growth factor significantly reduced Nrk expression. In addition, expression of Nrk was significantly reduced in regions of neointimal formation caused by guide-wire carotid artery injuries in mice, as well as in human atherosclerotic tissues, when compared to normal vessels. We identified that expression of matrix metalloproteinases (MMP3, MMP8 and MMP12) and inflammatory cytokines/chemokines (CCL6, CCL8, CCL11, CXCL1, CXCL3, CXCL5 and CXCL9) are synergistically induced by Nrk siRNA in LPS-treated mouse VSMCs. Moreover, we found that resveratrol significantly impaired LPS- and Nrk siRNA-induced expression of MMP3, CCL8, CCL11, CXCL3 and CXCL5. These results suggested that Nrk may play important roles in regulating pathological progression of atherosclerosis or neointimal- hyperplasia-related vascular diseases.

## INTRODUCTION

Atherosclerosis or neointimal hyperplasia refers to a pathological process of tunica intima thickening due to the proliferation and migration of vascular smooth muscle cells (VSMCs). Atherosclerosis is the major cause of myocardial infarction, ischemic stroke, ischemic gangrene, and peripheral vascular diseases [1–4]. As inflammatory disorders and immune dysregulation play important roles in promoting atherosclerosis and intimal hyperplasia, induction of cytokines and/or chemokines is involved at various stages of atherosclerosis [5]. Vascular remodeling by proteases, especially vascular matrix metalloproteinases (MMPs), is implicated in the progression of atherosclerosis [6–8]. Elevated expression of MMPs in vulnerable regions can be induced by cytokines in human atherosclerotic plaques [9, 10].

Nik-related kinase (Nrk), an X-linked protein kinase, is a member of the Ste20-type kinase family [11]. *Nrk* was first cloned from mice, and was initially detected in skeletal muscle during mouse embryogenesis [11]. Nrk (also known as NESK) contributes in activating the c-Jun N-terminal kinase (JNK) pathway in the late stages of murine embryogenesis [12], induces cofilin phosphorylation, and consequently enhances actin polymerization [13]. It has been reported that Nrk is essential for the regulation of trophoblast proliferation, placental development and fetoplacental induction of labor [14, 15]. Other than embryonic skeletal muscle and trophoblasts, Nrk is potentially expressed in human brain [16]. Moreover, Nrk deficiency during pregnancy results in the triggering of breast tumors in mice [17], and it has been shown that Nrk expression is positively correlated with survival in triple-negative breast cancer patients [18]. In this study, we aimed to assess the expression of Nrk in VSMCs, investigate its potential roles in regulating vascular inflammation, as well as elucidate clinical associations involving Nrk in atherosclerotic patients.

## RESULTS

### Expression of Nrk in VSMCs and mouse carotid artery

**Figure 1:**
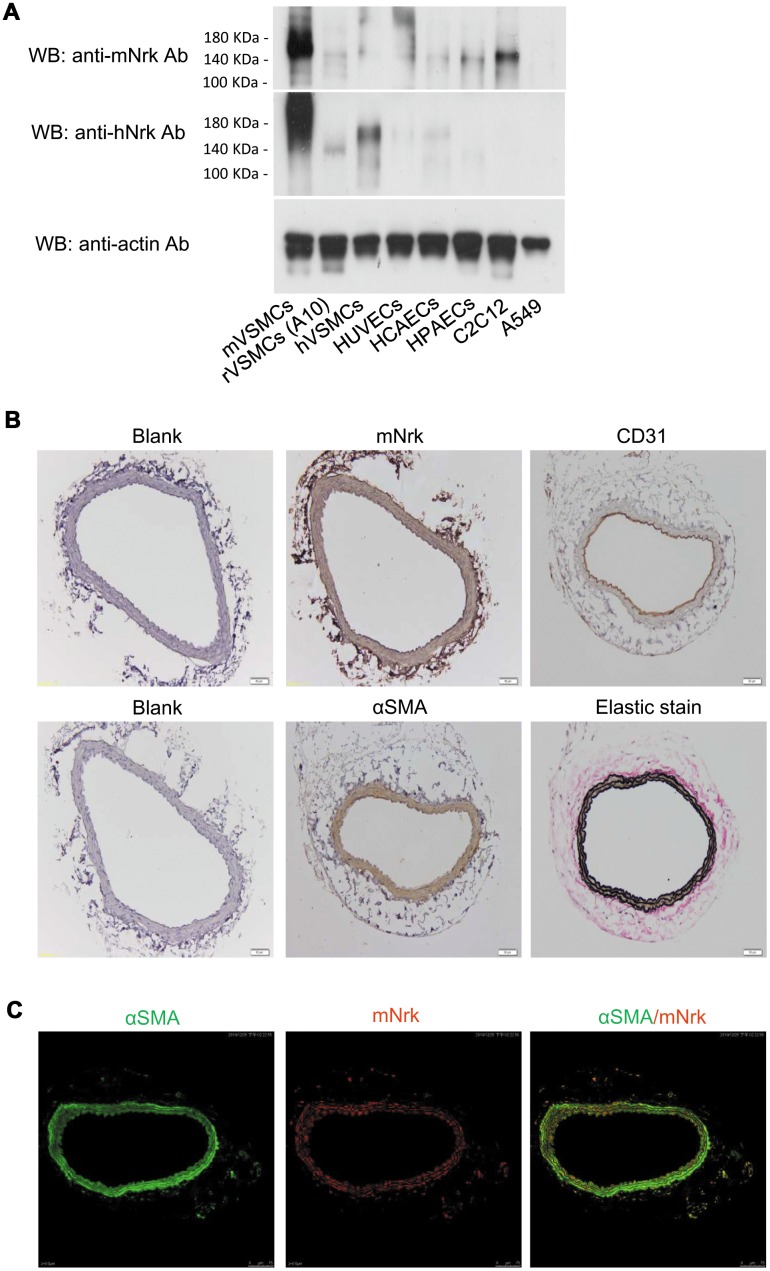
**Expression of Nrk in VSMCs.** (**A**) Expression of Nrk protein was determined by western blotting analysis in mVSMCs, rVSMCs (A10), hVSMCs, HUVECs, HCAECs, HPAECs, C2C12 and A549 cells. Primary antibodies against mNrk (upper panel) and hNrk (middle panel) were employed for the detection of Nrk. Actin was used as a loading control (lower panel). (**B**) Expression of mNrk in normal carotid artery of wild-type C57BL/6 mice was examined by immunohistochemical staining with primary antibodies against mNrk, CD31, αSMA, and elastic stain. Bar= 50 μM. (**C**) Expression and localization of αSMA (green) and mNrk (red) on mouse carotid artery was examined by double staining of immunofluorescence confocal microscopy.

An earlier report indicated that Nrk is expressed in embryonic muscle and trophoblast cells, but not in adult tissues or organs in mice [11]. To investigate whether Nrk is expressed in vascular cells, we examined the expression of mouse Nrk (mNrk) and human Nrk (hNrk) by western blot analysis of mouse VSMCs (mVSMCs), rat VSMCs (A10, rVSMCs), human VSMCs (hVSMCs), human umbilical vein endothelial cells (HUVECs), human coronary artery endothelial cells (HCAECs), human pulmonary artery endothelial cells (HPAECs), C2C12 (mouse myoblasts) and A549 cells (human lung adenocarcinoma). Expression of Nrk was abundant in mVSMCs, mid-range in hVSMCs and C2C12 cells, and low in rVSMCs, HUVECs, HCAECs and HPAECs (Figure 1A). As an internal negative control, expression of Nrk could not be detected in A549 cells (Figure 1A).

To further investigate whether Nrk is expressed in artery, mouse carotid artery and abdominal aorta were harvested and the expression of Nrk was examined by immunohistochemical (IHC) staining. Nrk was expressed in smooth muscle layers of carotid artery (Figure 1B) and abdominal aorta (Supplementary Figure 1). Staining of CD31 was performed as marker of endothelium, whereas αSMA and elastin stains were used as markers of smooth muscle layers (Figure 1B). Moreover, expression of Nrk in mVSMCs was further examined by immunofluorescence staining. Double staining of αSMA and mNrk was performed in mouse carotid artery (Figure 1C) and cultured VSMCs (Supplementary Figure 2) by confocal microscopy. Expression of mNrk (in red) was colocalized with αSMA (in green) in smooth muscle layers of carotid artery (Figure 1C, right panel) and VSMCs (Supplementary Figure 2, right panel).

### Reduced expression of Nrk in platelet-derived growth factor (PDGF) or lipopolysaccharide (LPS)-treated mVSMCs and arterial intimal hyperplasia in mice

**Figure 2:**
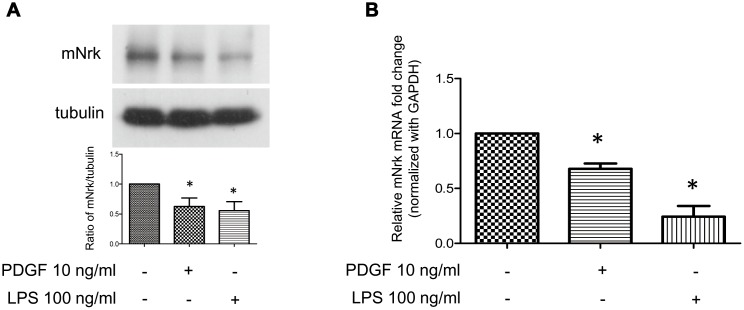
**Expression of Nrk was suppressed by PDGF and LPS in mVSMCs.** mVSMCs were serum starved (0.5% FBS in DMEM) for 24 h, followed by stimulation with PDGF (10 ng/ml) or LPS (100 ng/ml) for an additional 24 h. Expression of mNrk was determined by (**A**) western blotting (n=6) and (**B**) qPCR analysis (n=4). Gene expression of qPCR analysis results were normalized to both control cells as well as to *GAPDH*. Tubulin was used as a loading control for western blotting analysis. Scale bars: means ± SD. *, *p* <0.05.

**Figure 3:**
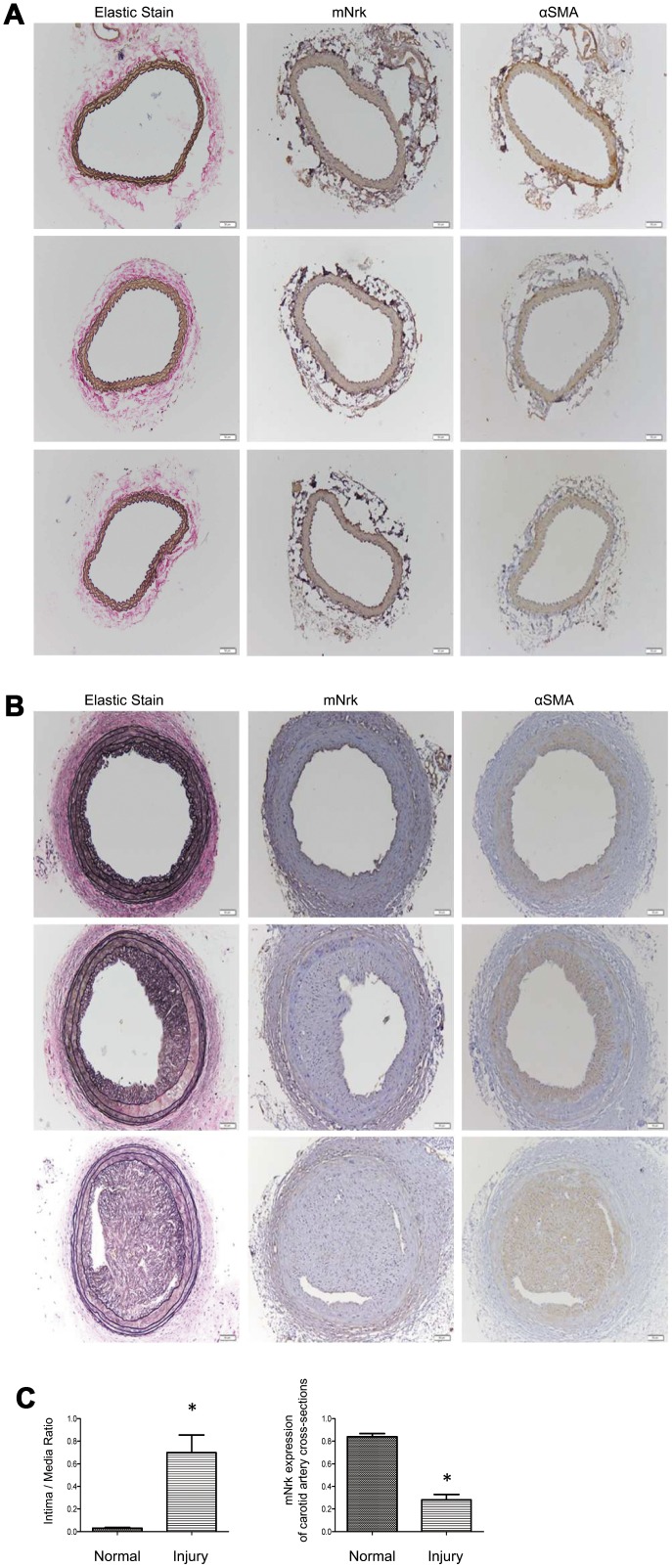
**Expression of mNrk, αSMA, and elastic staining in carotid artery of wild-type C57BL/6 mice subjected to guide wire injury for 4 weeks.** The expression of mNrk, αSMA, and elastic staining in three set of sections of (**A**) non-injured and (**B**) injured carotid arteries at 150-μm intervals was examined by immunohistochemical staining. Bar = 50 μM. (**C**) Left panel: Quantitation of intima/media (I/M) ratio (left panel, *p* = 0.00056) and Nrk expression (right panel, *p* = 1.184 × 10^-5^) in normal and injured carotid arteries. n = 9 for each group.

It has been demonstrated that treatment with PDGF or LPS triggers inflammatory responses, phenotypic switching from contractile to proliferative type of VSMCs, and generates inflammatory cytokines/chemokines, thereby promoting arterial atherosclerosis and venous neointimal hyperplasia [19–22]. To examine the effect of LPS or PDGF on Nrk expression, mVSMCs were treated with LPS (100 ng/ml) or PDGF (10 ng/ml) for 24 h, followed by examination of mNrk expression by western blot and qPCR analysis. LPS and PDGF significantly reduced mNrk expression in mVSMCs (Figure 2A, 2B). We further performed time course experiment for PDGF/LPS-treated mVSMCs by qPCR analysis. LPS and PDGF reduced mNrk at 24 and 48 hr but had no effect at 8 hr (Supplementary Figure 3). Intriguingly, the effect for reduced expression of mNrk in mVSMCs by LPS is more significant than PDGF (Figure 2 and Supplementary Figure 3). In addition, we found that reduced expression of mNrk by LPS/PDGF treatment was correlated with induced cell migration and proliferation of mVSMCs which was analyzed by trans-well assay (Supplementary Figure 4) and MTT analysis (Supplementary Figure 5). To investigate the expression of Nrk in an *in vivo* model, we performed a guide-wire injured-carotid artery experiment and examined expression of mNrk in the stenosis region of carotid artery by IHC analysis. The expression of mNrk in three set of sections of non-injured (Figure 3A) and injured (Figure 3B) carotid arteries at 150-μm intervals was examined. We found that mNrk expression was significantly lower in arterial intimal hyperplasia of injured mice when compared to normal arteries (Figure 3C).

### Expression of Nrk in human atherosclerotic tissues

**Figure 4:**
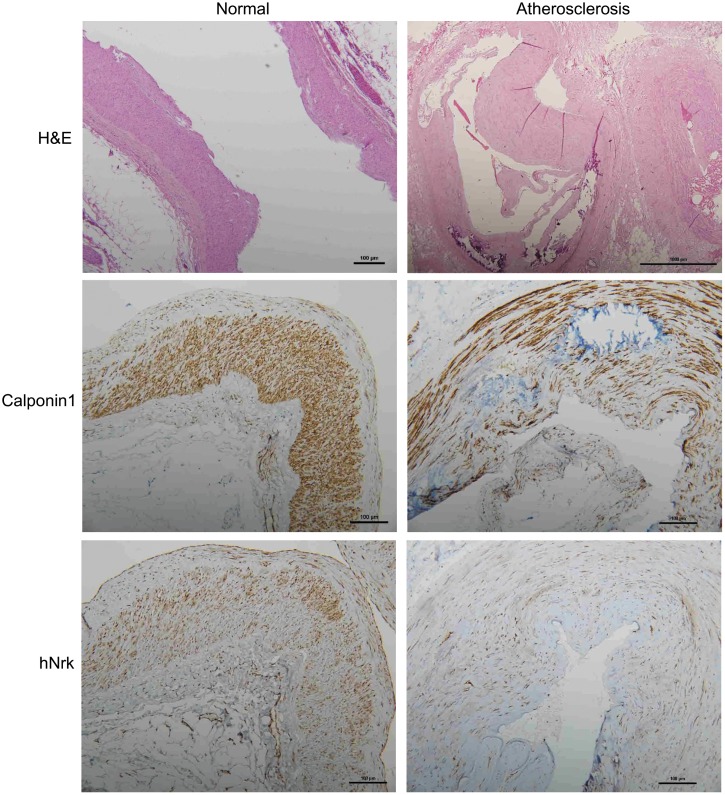
**Representative immunohistochemical staining of hNrk and calponin 1 in human normal (left panels) and atherosclerotic (right panels) vessels.** Bar = 100 μm.

To further investigate the expression of Nrk in atherosclerotic vessels, we examined the expression of Nrk in 47 human vessel samples (including 15 normal and 32 atherosclerotic vessels involving trauma or diabetic foot) by IHC staining. We found that Nrk was positively stained in smooth muscle regions of normal vessels (Figure 4, left panel). The expression of Nrk was significantly decreased in atherosclerotic regions (Figure 4, right panel) when compared to non-atherosclerotic areas. Moreover, quantified Nrk expression was correlated with clinicopathological characteristics including sex, age, diabetes status, hypertension, ischemic heart disease, and diagnosis groups (Table 1). Reduced Nrk expression was significantly associated with diabetes (*p* < 0.001), hypertension (*p* = 0.005), ischemic heart disease (*p* = 0.021) and atherosclerosis (*p* < 0.001) (Table 1). These results confirmed the finding that Nrk expression is reduced during the pathological progression of atherosclerosis or arterial intimal hyperplasia.

**Table 1:** Correlation of Nrk with clinicopathological parameters in patients with atherosclerosis.

**Parameter**	**Number (%)**	**NRK Q-score (Mean ± SD)**	**p-value**
All	47 (100%)	5.72±1.16	
Sex			0.510
Male	26 (55.3%)	5.77±1.34	
Female	21 (44.7%)	5.67±0.91	
Age			0.213
≤ 65 y/o	19 (40.4%)	5.95±1.22	
> 65 y/o	28 (59.6%)	5.57±1.10	
Diabetes			<0.001
No	16 (34.0%)	6.50±1.16	
Yes	31 (66.0%)	5.32±0.95	
Hypertension			0.005
No	15 (31.9%)	6.33±1.23	
Yes	32 (68.1%)	5.44±1.01	
Ischemic heart disease			0.021
No	23 (48.9%)	6.13±1.01	
Yes	24 (51.1%)	5.33±1.17	
Diagnosis group			<0.001
Normal	15 (31.9%)	7.00±0.00	
Atherosclerosis	32 (68.1%)	5.13±0.91	

### Regulation of MMPs and chemokines by Nrk in VSMCs

**Figure 5:**
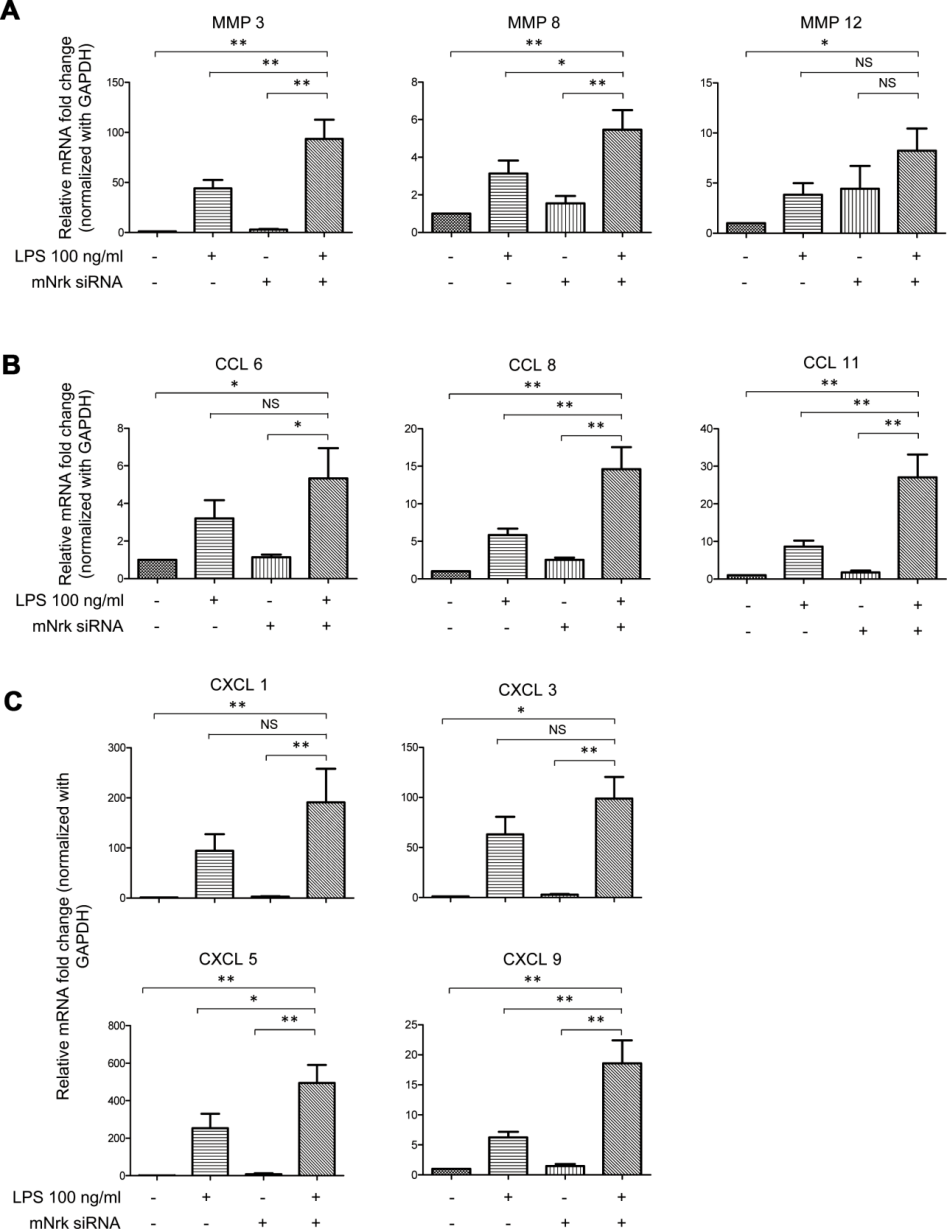
**Expression of MMPs and chemokines in LPS- and Nrk-siRNA treated mVSMCs.** mVSMCs were serum starved (0.5% FBS in DMEM) for 24 h and then treated with LPS (100 ng/mL) for 24 h. Cells were further transfected with 20 nM of negative control or mNrk siRNA for an additional 48 h. Expression of (**A**) *MMP3* (n=13)*, MMP8* (n=13) and *MMP12* (n=14); (**B**) *CCL6* (n=14)*, CCL8* (n=14) and *CCL11* (n=14); (**C**) *CXCL1* (n=14)*, CXCL3* (n=11)*, CXCL5* (n=10) and *CXCL9* (n=13) was determined by qPCR. Gene expression results of qPCR analysis were normalized to both control cells as well as *GAPDH*. Scale bars: means ± SD. *, *p* < 0.05, **, *p* < 0.01, ***, *p* < 0.001.

To investigate potential downstream regulators modulated by Nrk, we performed gene expression profiling by microarray analysis in Nrk siRNA- and LPS-treated mVSMCs (Supplementary Tables 1 and 2). Knockdown efficiency of Nrk in mVSMCs was confirmed by western blotting and qPCR (Supplementary Figure 6), and the results of mRNA expression quantitation from microarray analysis were validated by qPCR. Intriguingly, we found that several inflammation regulatory factors, including MMPs and chemokines, were induced by either Nrk siRNA or LPS in mVSMCs. Silencing of Nrk and LPS treatment synergistically increased MMP3, MMP8, MMP12, CCL6, CCL8, CCL11, CXCL1, CXCL3, CXCL5 and CXCL9 expression (Figure 5). Although it was not statistically significant, expression of IL-6 was increased in Nrk siRNA and LPS-treated mVSMCs (Supplementary Figure 7). These results suggested that Nrk may play an anti-inflammatory role during the pathological progression of arterial intimal hyperplasia by suppression of inflammatory factors and proteases.

### Effect of resveratrol on LPS- and Nrk siRNA-induced MMPs and chemokines in VSMCs

**Figure 6:**
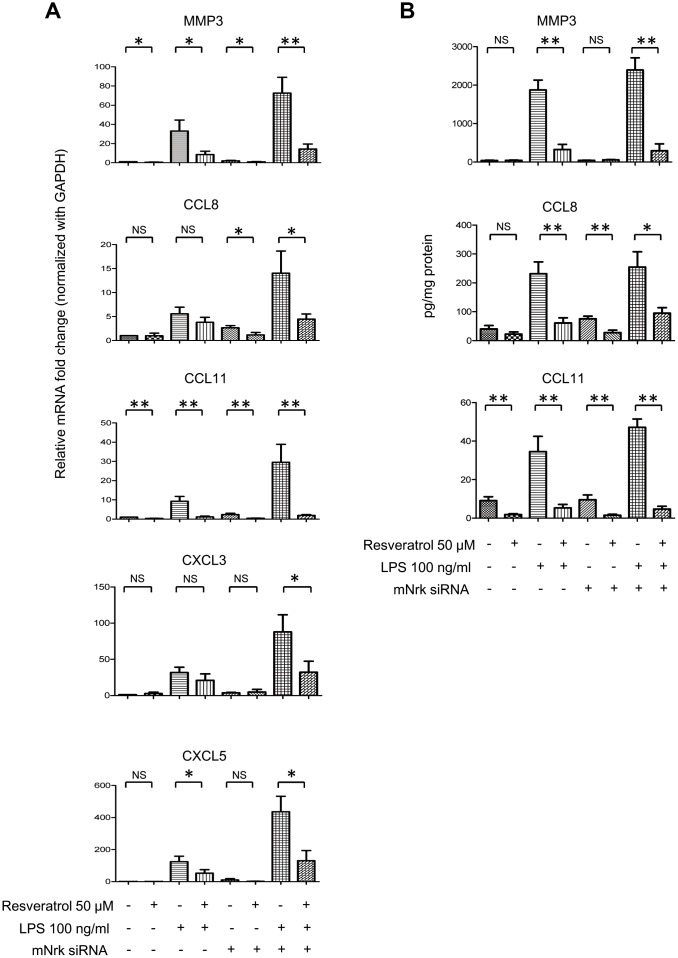
**Effect of resveratrol on LPS- and Nrk siRNA-stimulated MMPs and chemokines.** (**A**) mVSMCs were serum starved (0.5% FBS in DMEM) for 24 h and then treated with LPS (100 ng/mL) and/or resveratrol (50 μM) for 24 h. Cells were further transfected with 20 nM of negative control or mNrk siRNA for an additional 48 h. Expression of (**A**) *MMP3* (n=8)*, CCL8* (n=8)*, CCL11* (n=8)*, CXCL3* (n=6) and *CXCL5* (n=6) was determined by qPCR. Gene expression results of qPCR analysis were normalized to both control cells as well as *GAPDH*. (**B**) Protein levels of MMP3 (n=7), CCL8 (n=6) and CCL11 (n=7) in cultured conditioned media were determined by ELISA (normalized to total protein concentration). Scale bars: means ± SD. *, *p* < 0.05, **, *p* < 0.01, ***, *p<*0.001.

It has been reported that resveratrol exhibits cardiovascular protective effects by suppressing oxidative stress and inflammation [23, 24]. To investigate the effect of resveratrol on Nrk-regulated downstream gene expression, we performed experiments to elucidate the effects of resveratrol on Nrk silencing-induced inflammatory factors. The expression of *MMP3, CXCL3, CXCL5, CCL8* and *CCL11* was determined by qPCR in resveratrol-treated mVSMCs, combined with Nrk siRNA- and/or LPS treatment. Resveratrol significantly abrogated the expression of *MMP3*, *CXCL3, CXCL5, CCL8* and *CCL11* induced by Nrk siRNA- and/or LPS (Figure 6A). Because MMP3, CCL8 and CCL11 have been implicated as being involved in human atherosclerotic progression [8–10, 25–29], we confirmed the production of MMP3, CCL8 and CCL11 in the culture medium of mVSMCs by ELISA analysis. LPS and Nrk silencing synergistically induced MMP3, CCL8 and CCL11 in the cultured media, whereas resveratrol attenuated the effect induced by LPS- and Nrk siRNA (Figure 6B). These results indicated that resveratrol may play an anti-inflammatory role by impairing Nrk silencing-induced production of chemokines and MMPs.

## DISCUSSION

Nrk belongs to the Ste20-type kinase family, which is believed to contribute to regulating the MAPK cascade [11, 12]. It has been reported that Nrk is required for placental development, and Nrk regulates trophoblast proliferation through modulating Akt phosphorylation [14, 15]. However, the expression of Nrk in other organs or tissues, substrates of Nrk, molecular mechanisms for regulating its kinase activity, as well as regulated signaling cascades, have never been elucidated. In this study, we found that Nrk is expressed in normal VSMCs (Figure 1) and that its expression is significantly reduced in neointimal and atherosclerotic regions in murine and human arteries (Figures 3 and 4). We have further reported that reduced Nrk expression was associated with induction of MMPs and chemokines in VSMCs (Figure 5). The mechanisms involved in molecular regulation remain unclear, and further investigation is needed to elucidate how reduced Nrk expression results in MMP induction and chemokine production in VSMCs.

An *MMP3* (also known as stromelysin) promoter 5A/6A polymorphism was reported to involve the regulation of promoter activity and gene expression [30, 31]. The *MMP3* promoter polymorphism and expression levels of circulating MMP3 in patients are associated with acute myocardial infarction, left ventricle dysfunction, ischemic stroke, hemorrhagic stroke, and coronary syndrome [32–39]. MMP3 is considered to be a potential marker to predict long-term risk of cardiovascular diseases. In this study, we found that expression of MMP3 is synergistically induced by Nrk silencing and LPS (Figure 5A). We hypothesized that Nrk may plays important roles in suppressing arterial inflammation and atherosclerotic progression.

CCL8 and CCL11 are CC chemokines and potent chemoattractants. CCL8 (monocyte chemoattractant protein-2, MCP-2) selectively binds to and activates the chemokine receptor CCR2, and levels of circulating CCL8 as well as other chemokines are significantly associated with risk of atherosclerotic cardiovascular disease [25, 26]. Moreover, increased expression of CCL11 (eotaxin) and CCR3 receptors is found in human atherosclerosis [28]. CCL11 is reported to augment calcification in VSMCs [40], and is associated with coronary transluminal angioplasty [41]. Moreover, CCL11 is abundantly expressed in smooth muscle cells (SMCs) of atherosclerotic plaque and injured arteries [42], and CCL11 induces murine SMC migration through a CCR3-dependent mechanism [42]. Our findings indicated that expression of CCL8, CCL11 and several chemokines was regulated by Nrk in VSMCs (Figure 5B, 5C). Further investigation is needed to elucidate how these chemokines are regulated by Nrk.

Resveratrol is a phytoalexin found in various plants, including grape and berry [43]. Increasing evidence has demonstrated that resveratrol exhibits anti-inflammatory effects, thereby impairing proliferation of VSMCs and attenuating atherosclerosis and restenosis [44–46]. Here, our results revealed that resveratrol attenuated expression of MMP3, CCL8 and CCL11 induced by LPS- and Nrk silencing in mVSMCs (Figure 6A, 6B). Intriguingly, resveratrol had no significant effect on the expression level of mNrk of mVSMCs (Supplementary Figure 8). These results suggested that resveratrol or other anti-inflammatory compounds may play protective roles in terms of vascular function through modulating Nrk activity and/or its downstream factors. Our finding will enable further investigation into uncovering the regulating mechanism.

Earlier studies indicated that Nrk is expressed in embryonic muscle and trophoblast cells, but not in any adult tissues or organs in mice [11, 14]. However, the expression and physiological function of Nrk in humans has never been elucidated. It has been reported that Nrk regulates trophoblast proliferation and placental development through modulating Akt signaling [15]. Moreover, JNK pathway was demonstrated as the downstream regulator of Nrk during the late stage of embryogenesis [12]. Thus, Akt, MAPKs or other signal pathways are potential downstream targets and effectors of Nrk in regulating cellular and physiological functions. In this study, we aimed to examine the expression of Nrk in arterial intima and investigate its role in atherosclerosis or intimal hyperplasia. Our current results revealed that Nrk is abundantly expressed in VSMCs, and attenuates inflammation and pathological progression of neointimal formation through suppressing MMPs and chemokine production by VSMCs. Further studies are needed to assess the signal pathway underlying Nrk-mediated regulation of MMPs and inflammatory chemokines.

To our knowledge, this is the first finding that Nrk is expressed in intimal regions of both normal human and murine vessels (Figures 1, 3 and 4). Decreased expression of Nrk in arterial VSMCs is associated with diabetes and cardiovascular diseases in atherosclerotic patients (Table 1). Thus, Nrk may plays an important role in suppressing expression of inflammatory factors in VSMCs. Developing approaches targeting Nrk and its downstream factors may be a potential strategy for the prevention of intimal hyperplasia and related atherosclerotic heart diseases.

## MATERIALS AND METHODS

### Reagents

Dimethyl sulfoxide (DMSO) and lipopolysaccharides (LPS) were purchased from Sigma-Aldrich Chemical Company (St. Louis, MO, USA). Dulbecco’s modified Eagle’s medium (DMEM), fetal bovine serum (FBS), MEM non-essential amino acids (NEAA), penicillin and streptomycin were obtained from Invitrogen-Gibco (Grand Island, NY, USA). Recombinant human PDGF-BB was purchased from PeproTech, Inc. (Rocky Hill, NJ, USA). Resveratrol was sourced from Cayman Chemical Company (Ann Arbor, Michigan, USA).

### Cell culture and siRNA transfection

Primary mVSMCs were isolated from mouse aortas and cultured in DMEM [47, 48]. rVSMCs A10 (Bioresource Collection and Research Center, Hsinchu, Taiwan); hVSMCs, HUVECs, C2C12 and A549 (American Type Culture Collection, Manassas, VA, USA); HCAECs and HPAECs (Lonza, Walkersville, MD, USA) were maintained as described previously [49]. Silencing experiments were performed by transfection with siRNA of mNrk (Sequences: GGACCAAGAACUUCAACAATT; UUGUUGAAGUUCUUGGUCCTT) and negative control (Sequences: UUCUCCGAACGUGUCACGUTT; ACGUGACACGUUCGGAGAATT) (Shanghai GenePharma, Shanghai, China) in mVSMCs using Lipofectamine RNAiMAX (Thermo Fisher Scientific, Victoria, Australia).

### Western blot analysis

Cells were harvested and lysed in ice-cold RIPA buffer (Millipore, Temecula, CA, USA) containing cocktail protease inhibitors (Roche, IN, USA). Cell lysates were centrifuged at 15,000 rpm for 20 minutes at 4°C, and protein concentration was determined using a Bio-Rad protein assay kit (Bio-Rad Laboratories, CA, USA). Equal amounts of protein from each sample were run through a gradient SDS-PAGE gel, followed by immunoblotting onto PVDF membranes. The membranes were blocked then probed with primary antibodies against actin (Sigma-Aldrich, St. Louis, MO, USA), tubulin (Sigma-Aldrich, St. Louis, MO, USA), hNrk (MyBioSource Inc., San Diego, USA), and mNrk (custom made, sequence: MSARKTPLPEIGRRC). The membranes were immersed in 0.1% PBST containing horseradish peroxidase-conjugated secondary antibody and protein levels were determined by use of enhanced chemiluminescence reagents.

### Quantitative real-time PCR (qPCR)

Total RNA was extracted using RNAzol^®^ RT (RN190, Molecular Research Center, Cincinnati, OH, USA) and cDNAs were synthesized using the PrimeScript RT reagent kit (TAKARA Bio Inc., Kusatsu-shi, Japan). qPCR analysis was performed using SYBR Green (Kapa Biosystems, Woburn, MA, USA) with specific oligonucleotide primers (Supplementary Table 3) on an ABI Prism 7900 System (ABI Applied Biosystems, Waltham, Massachusetts, USA). Levels of specific gene transcripts were normalized to *GAPDH* in the same sample.

### Carotid artery injury operation

The protocol of this study was performed in accordance with guidelines and regulations approved by the Institutional Animal Care and Use Committee of the National Health Research Institutes. 8- to 10-week-old C57BL/6 mice were purchased from the National Laboratory Animal Center of Taiwan and housed in pathogen-free microisolator cages. To achieve carotid artery injuries, a mouse wire injury model was performed [50–52]. Throughout all procedures, the level of sedation of the animal was routinely checked, and all exposed tissues were kept moist using sterile saline. All untied sutures were removed, and tissue and skin incisions closed.

### Histological and immunohistochemical analysis

Carotid arteries were harvested on day 28 and fixed in 10% formaldehyde. Paraffin cross-sections were prepared at a thickness of 5 μm and stained with antibodies against CD31 (Abcam, Cambridge, UK), alpha smooth muscle Actin (αSMA, Sigma-Aldrich), hNrk (MyBioSource Inc.), mNrk (custom made, sequence: CLNNDPKSKKRQKAM) and calponin 1 (Santa Cruz Biotechnology, Dallas, Texas, USA) followed by the N-Histofine^®^ MOUSESTAIN KIT (Nichirei Biosciences Inc., Tokyo, Japan) and Polink-2 Plus HRP anti Rabbit Detection Kit (GBI Labs, Mukilteo, WA, USA). At least three sets of sections at 150-μm intervals were used for morphometry of each arteries. Digitized images of H&E and elastic staining (Elastic Stain Kit, Sigma-Aldrich) were analyzed using Image-Pro Plus 6.0 (Media Cybernetics, USA) to calculate the intimal area to the medial area ratio (I/M).

### Immunofluorescence staining

Immunofluorescence staining was performed as described previously [53, 54]. Series sections of carotid arteries and mVSMCs were fixed with 2% paraformaldehyde and permeabilized with 0.1% Triton X-100. After blocked with PBS containing 10% FBS. Fixed sections and mVSMCs were incubated with the primary antibodies of anti-αSMA (A5228; Sigma) and anti mNrk (custom made from GenScript) (Sequences: CLNNDPKSKKRQKAM) in PBS containing 1% FBS at 4°C overnight, followed by incubation with Alexa Fluor® 488 and Alexa Fluor® 594 secondary antibody (Jackson ImmunoResearch Inc., West Grove, PA, US) in PBS containing 5% bovine serum albumin at room temperature for additional 2 hrs. Samples were mounted and images were analyzed by use of the Leica TCS SP5 Confocal Imaging System (Leica, Germany).

### Migration assay

Bio-coat cell migration Boyden chambers were used to perform cell migration assay [53, 54] of mVSMCs (Becton Dickinson, Pont-de-Claix, France). Briefly, mVSMCs were trypsinized and suspended in 0.1% BSA-DMEM and added to the upper wells. mVSMCs were allowed to migrate toward the bottom wells containing 10% FBS-DMEM for 6 hours. mVSMCs remaining on the upper side were removed, and migrated cells on the bottom side were fixed and stained with 0.1% crystal violet containing 20% ethanol and 1% formaldehyde for 20 minutes. Cell migration was quantified by counting the total number of migrated cells in each well.

### Cell proliferation analysis

Cells were seeded into 96-well plates and incubated with 0.5% FBS medium containing the indicated concentrations of PDGF or LPS for 24 h or 48 h. MTT was added to each well and incubated at 37 °C for 3 h. Subsequently, the yellow MTT solution was removed, and 200 μL of dimethyl sulfoxide was added. The absorbance at 570 nm was measured with a reference wavelength of 690 nm.

### Patients and specimens

A series of 47 patients with trauma or diabetic foot ulcer and undergoing amputation of foot or leg at Taichung Veterans General Hospital, Taichung, Taiwan, were enrolled. Further examination of resected specimens confirmed the diagnosis of normal (16) or atherosclerosis (31) of vessels. The de-identified specimens and clinical information were collected from Specimen-Bank, and this study was approved by the Institutional Review Board of Taichung Veterans General Hospital.

### Immunohistochemical analysis of human samples

Immunohistochemical staining was carried out on paraffin-embedded sections with an automatic immunostaining device and Optiview detection kit (Ventana XT Medical Systems, Tucson, AZ, USA). Antigen retrieval was carried out automatically by the device, according to the manufacturer's instructions (BenchMark XT Roche, NJ, USA). Expression of hNrk in tissue sections was analyzed using an Nrk antibody from MyBioSource Inc. Antigen retrieval reagent: SCC1 at 37°C for 32 minutes. For detection, Optiview DAB IHC Detection Kit from Ventana Medical Systems was used. Scoring of each sample was made independently and blindly by two pathologists. Intensity of immunostaining was scored semi-quantitatively using a Quick score (Q-score) method based on intensity and heterogeneity [53–58]. Briefly, the staining intensity was scored as 0 (negative), 1 (weak), 2 (moderate), or 3 (strong). For heterogeneity staining, the proportion of tumor cells stained for hNrk was scored as 0, 1 (1%-25%), 2 (26%-50%), 3 (51%-75%), or 4 (76%-100%). The Q-score of a given tissue sample was the sum of the intensity and heterogeneity scores, and ranged from 0 to 7. Those rare cases with < 5% weakly stained tissue were considered negative.

### Microarray analysis

Gene expression profiles of LPS- and mNrk siRNA-transfected mVSMCs were analyzed using microarray analysis (Affymetrix Mouse Gene 2.0 ST array, Affymetrix Inc., Santa Clara, CA, USA) according to the manufacturer’s recommendations.

### ELISA analysis

Protein levels of MMP3, CCL8 and CCL11 in the culture medium were determined by sandwich ELISA (R&D Systems Inc., Minneapolis, MN, USA) in 96-well microtiter plates coated with anti-mouse MMP3, CCL8, and CCL11 in PBS overnight. After the plates were blocked and washed, diluted samples or standards were added, and the plates were incubated for 2 h. The detection antibody was incubated for 2 h and streptavidin-HPR was incubated for 20 min. The 3,3’,5,5’-tetramethylbenzidine (TMB) liquid substrate solution was added and incubated in the dark for 10 min. The color reaction was arrested by adding stop solution. Color intensity was measured using a spectrophotometer at a wavelength of 450 nm.

### Statistical analysis

All statistical analysis for the correlation of Nrk with clinicopathological parameters in patients with atherosclerosis was done by Mann-Whitney U test. Student’s *t*-test was used to analyze differences between groups. For pairwise comparison on relative Western blots, mRNA expression and intima/media ratio, Mann-Whitney U test was also used. In identifying the effects of LPS treatment and mNrk siRNA effects on the mRNA expression of various inflammatory cytokines, Kruskal-Wallis one-way ANOVA with post-hoc Dunnett’s tests were used to comparing differences between groups. *P* values < 0.05 were considered statistically significant.

## Supplementary Material

Supplementary Figures

Supplementary Tables
