# Supplementary material for: Expression of Nik-related kinase in smooth muscle cells attenuates vascular inflammation and intimal hyperplasia
Source: Aging (Albany NY). 2020 Apr 24;12(8):7511–33. doi: 10.18632/aging.103104 (PMC7202544; doi:10.18632/aging.103104)
Supplement: Supplementary Figures [file aging-12-103104-s002..pdf]

SUPPLEMENTARY FIGURES

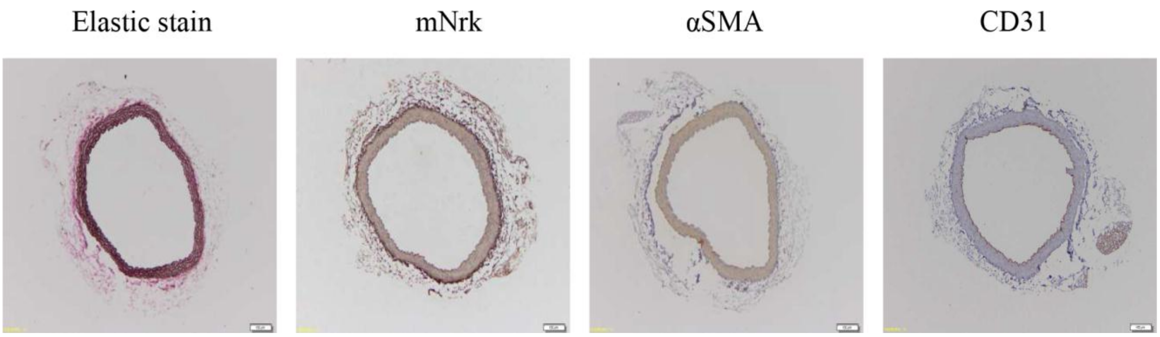

Supplementary Figure 1. Expression of mNrk in normal abdominal aorta of wild-type C57BL/6 mice was examined by immunohistochemical staining with primary antibodies against mNrk, CD31, αSMA and elastic stain. Bar=50 μM.

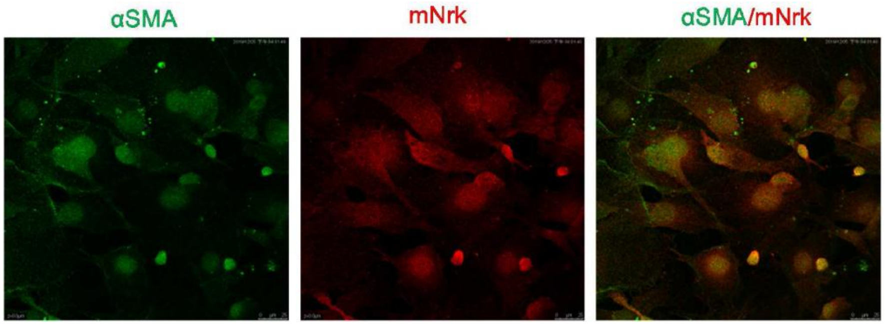

Supplementary Figure 2. Expression and localization of αSMA (green) and mNrk (red) on VSMCs was examined by double staining of immunofluorescence confocal microscopy.

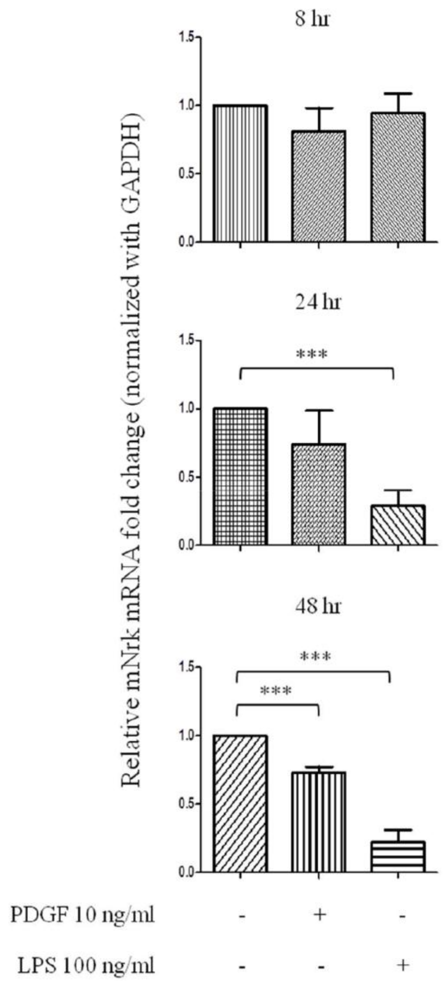

Supplementary Figure 3. Expression of mNrk in PDGF and LPS-treated mVSMCs at 8 hr, 24 hr and 48 hr was determined by qPCR (n=4). Gene expression results of qPCR analysis were normalized to both control cells as well as *GAPDH*. Scale bars: means ± SD. ***, *p* < 0.001.

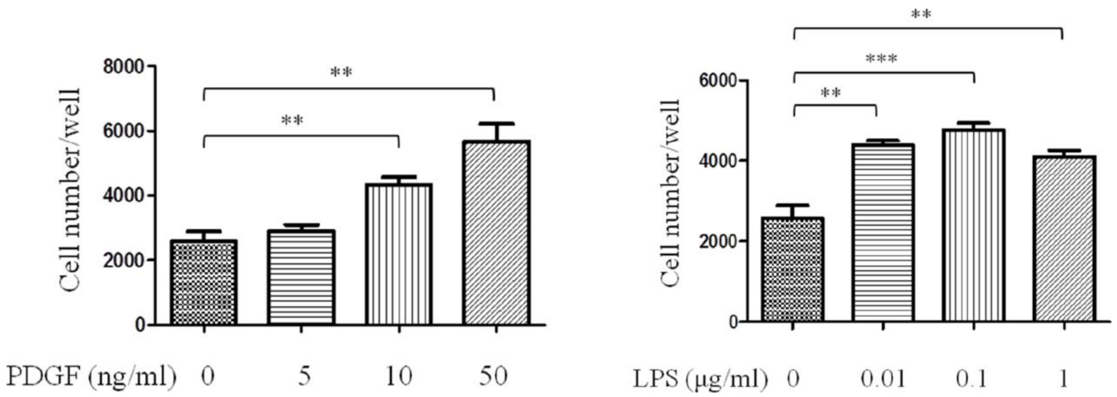

Supplementary Figure 4. Cell migration of mVSMCs were analyzed by Boyden chambers trans-well assay. mVSMCs were treated with PDGF and LPS at indicated concentrations and allowed to migrate toward the bottom wells for 6 hours. Cell migration was quantified by counting the total number of migrated cells in each well (n=4). Scale bars: means ± SD. ***p*<0.01, ****p*<0.001.

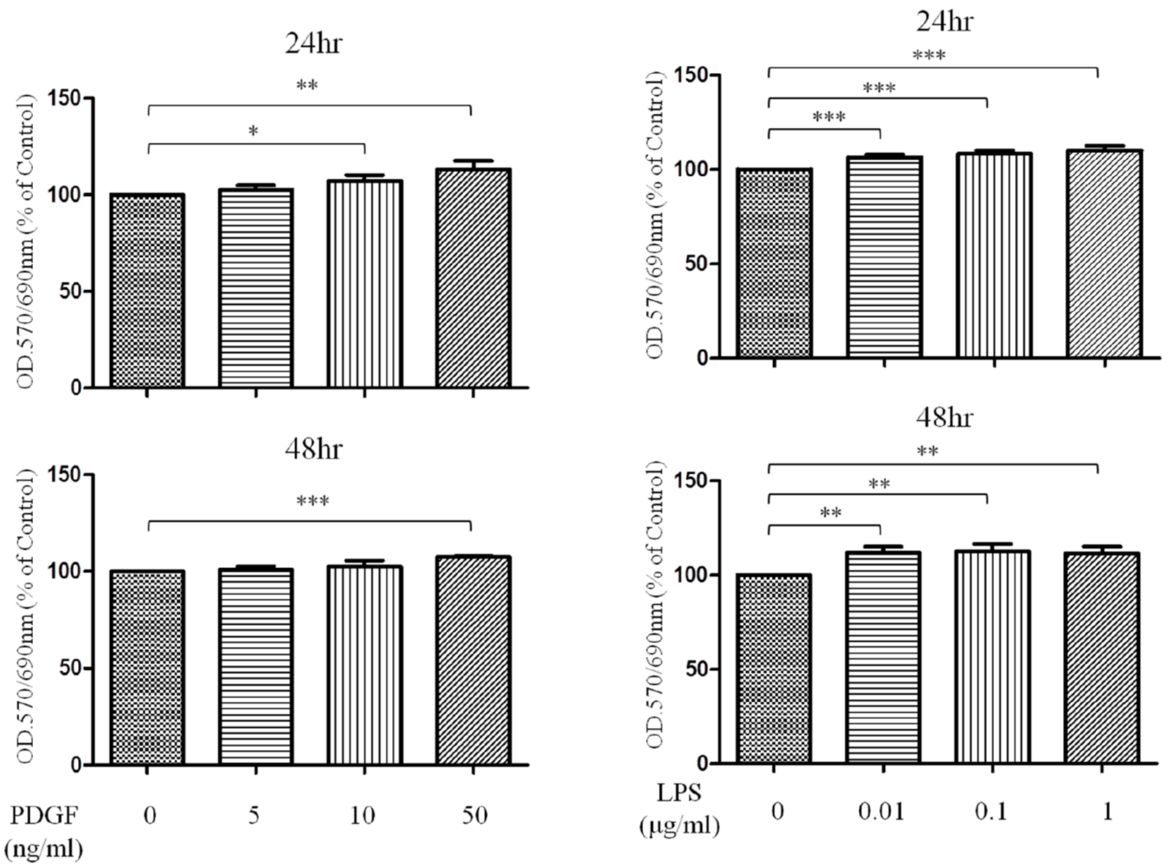

Supplementary Figure 5. mVSMCs were treated with PDGF and LPS at indicated concentrations for 24 hr and 48hr. Cell proliferation of mVSMCs were analyzed by MTT analysis (n=5). Scale bars: means ± SD. *p<0.05, **p<0.01, ***p<0.001.

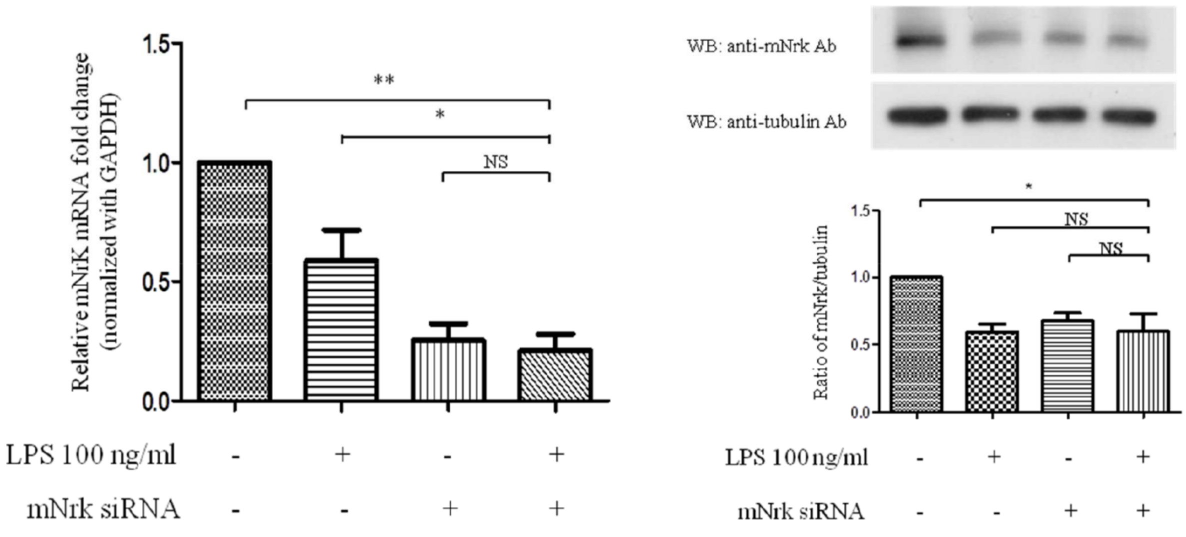

Supplementary Figure 6. Expression of mNrk was determined by qPCR (left, n=11) and western blotting (right, n=4) analysis in LPS and/or mNrk siRNA treated VSMCs. mVSMCs were serum starved (0.5% FBS in DMEM) for 24 h and then treated with LPS (100 ng/mL) for 24 h. Cells were further transfected with 20 nM of negative control or mNrk siRNA for an additional 48 h. Relative mNrk mRNA expression was determined by qPCR analysis (normalized to control cell as well as *GAPDH*), *P<0.05, **P<0.01. Tubulin was used as loading control.

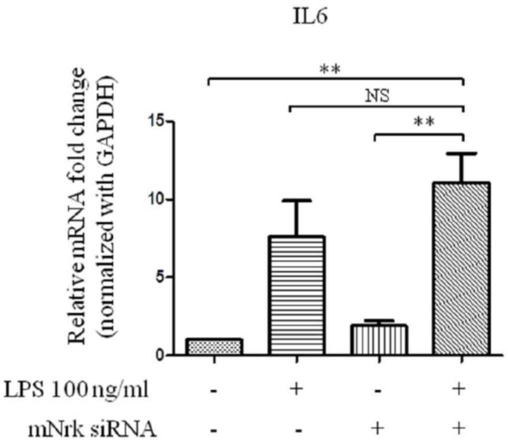

Supplementary Figure 7. Expression of IL-6 was determined by qPCR analysis in LPS and/or mNrk siRNA treated VSMCs (n=14). mVSMCs were serum starved (0.5% FBS in DMEM) for 24 h and then treated with LPS (100 ng/mL) for 24 h. Cells were further transfected with 20 nM of negative control or mNrk siRNA for an additional 48 h. Relative IL-6 mRNA expression was determined by qPCR analysis (normalized to control cell as well as *GAPDH*), NS, not significant, **P<0.05.

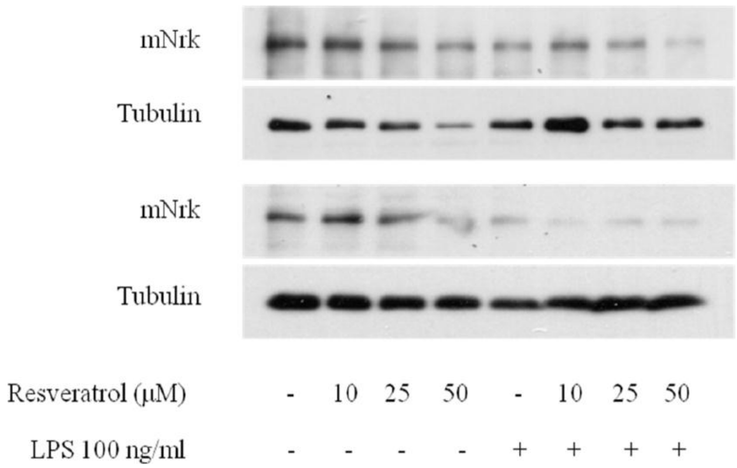

Supplementary Figure 8. Two representative expressions of mNrk examined by western blotting analysis in resveratrol and/or LPS VSMCs. mVSMCs were serum starved (0.5% FBS in DMEM) for 24 h and then treated with resveratrol (at indicated concentrations) and/or LPS (100 ng/mL) for 24 h. Tubulin was used as loading control.
