# Supplementary material for: Expression of Nik-related kinase in smooth muscle cells attenuates vascular inflammation and intimal hyperplasia
Source: Aging (Albany NY). 2020 Apr 24;12(8):7511–33. doi: 10.18632/aging.103104 (PMC7202544; doi:10.18632/aging.103104)
Supplement: Supplementary Tables [file aging-12-103104-s001..pdf]

SUPPLEMENTARY TABLES

Supplementary Table 1. LPS and/or Nrk siRNA induced genes analyzed by microarray.

		Increase (Fold)					
Gene symbol	RefSeq	LPS vs. CTR	NrksiRNA* LPS vs. LPS	Gene symbol	RefSeq	LPS vs. CTR	NrksiRNA* LPS vs. LPS
Cxcl3	NM_203320	3.20	5.98	Slc16a2	NM_009197	1.76	2.04
Cfb	NM_008198	2.91	5.97	Sp140	NM_001013817	1.67	2.04
Mmp8	NM_008611	1.70	5.52	Cybb	NM_007807	1.72	1.98
Serpina3n	NM_009252	2.62	5.21	Serpina3f	NM_001168294	9.63	1.97
Ism1	NM_001126490	2.13	4.65	Gm4902	NM_001164327	2.45	1.96
Cxcl5	NM_009141	52.51	4.54	Marco	NM_010766	4.22	1.95
Ccl6	NM_009139	1.95	4.29	Tlr2	ENSMUST00000029623	2.79	1.93
Fpr1	NM_013521	2.92	4.08	Serpina3i	NM_001199940	6.50	1.92
Pf4	NM_019932	1.75	3.93	Gm15987	NR_045009	3.59	1.90
Ptx3	NM_008987	1.98	3.69	Il1rl1	ENSMUST00000097772	2.54	1.89
Ppbp	NM_023785	2.00	3.63	Il1r1	NM_008362	1.80	1.88
Mmp12	NM_008605	1.70	3.61	Slc2a6	NM_172659	1.50	1.87
Trpc3	NM_019510	1.76	3.50	Vdr	NM_009504	2.13	1.86
Zbp1	NM_021394	2.09	3.40	BC061237	BC061237	1.52	1.84
Il13ra2	ENSMUST00000033646	3.70	3.13	Tspan11	ENSMUST00000032501	1.64	1.83
Tgfbr3	ENSMUST00000031224	1.83	3.11	Kcnn3	NM_080466	2.99	1.82
Angpt1	ENSMUST00000022921	2.54	3.03	Rassf4	NM_178045	1.61	1.82
Kng2	NM_201375	1.71	2.91	Itih5	NM_172471	1.58	1.81
Cxcl1	NM_008176	12.22	2.81	Gm11428	NM_001081957	2.50	1.79
Ptgfr	ENSMUST00000029670	1.79	2.81	Tarm1	NM 177363	2.15	1.79
Abcd2	NM_011994	1.65	2.79	Hp	NM_017370	16.02	1.76
Cyp7b1	NM_007825	2.95	2.75	Prr16	ENSMUST00000097576	2.84	1.75
Pdgfra	NM_001083316	2.68	2.68	Bdkrb1	NM_007539	1.69	1.74
Mmp3	NM_010809	18.35	2.66	Ccbe1	NM_178793	1.63	1.72
BC022960	BC022960	1.52	2.55	Ptch1	NM_008957	1.70	1.71
Irg1	ENSMUST00000022722	8.25	2.51	LOC100504873	XM_003689344	1.88	1.69
Gpm6b	NM_001177956	6.13	2.50	Csprs	NM_033616	2.08	1.63
Stfa3	NM_025288	3.04	2.50	Csprs	NM_033616	2.08	1.63
Clec4e	NM_019948	5.91	2.48	Gm7609	NM_001081746	2.09	1.63
Cd59b	ENSMUST00000111130	1.51	2.45	Olfr 1316	NM_146742	1.85	1.62
Cxcl9	NM_008599	2.47	2.44	Lcn2	NM_008491	59.96	1.61
Emrl	NM_010130	1.65	2.37	Ccl9	NM_011338	2.25	1.61
Clrb	NM_001113356	1.93	2.34	A4galt	NM_001170954	2.33	1.60
Pi15	NM_053191	1.88	2.30	Csprs	NM_033616	1.63	1.59
Clec4a1	NM_199311	2.55	2.29	Gfpt2	NM_013529	1.82	1.58
Draml	NM_027878	4.04	2.27	Il6	NM_031168	7.90	1.57
Pde7b	NM_013875	3.61	2.23	Map3k8	NM_007746	1.64	1.57
Stcl	ENSMUST00000014957	2.13	2.23	Rbp1	NM_011254	1.66	1.57
Fgf7	NM_008008	1.52	2.20	Fgf10	NM_008002	2.51	1.56
Ccl8	NM_021443	1.91	2.16	Pcx	NM_001162946	1.55	1.56
Gdpd2	NM_023608	2.44	2.15	Nfkbia	NM_010907	2.51	1.55

Epas1	NM_010137	1.83	2.13	Pion	NM_175437	1.67	1.53
Mme	NM_008604	2.68	2.08	Serpina3c	ENSMUST00000085050	2.18	1.53
Serpina3h	NR_033450	5.50	2.07	Tnfsf13b	NM_033622	2.02	1.52
Gm9041	NM_001244651	1.79	2.07	$^{G}yp^{c}$	NM_001048207	1.68	1.51
Ccl11	NM_011330	2.96	2.06	Ppp1r3c	ENSMUST00000087321	1.56	1.51
Cbr2	ENSMUST00000026148	1.56	2.05				

Supplementary Table 2. LPS and/or Nrk siRNA reduced genes analyzed by microarray.

Gene symbol	RefSeq	LPS vs. CTR	NrksiRNA* LPS vs. LPS	Gene symbol	RefSeq	LPS vs. CTR	NrksiRNA* LPS vs. LPS
Nrk	NM_013724	-2.26	-3.75	Chac1	ENSMUST00000028780	-1.57	-1.57
Prex2	NM_029525	-1.52	-3.47	Ptprb	NM_029928	-1.74	-1.57
Ccdc141	ENSMUST00000049544	-1.93	-2.23	D3Bwg0562e	NM_177664	-1.55	-1.56
Rarres1	NM_001164763	-1.69	-2.15	Gm5091	NR_046164	-1.51	-1.56
Lama3	NM_010680	-2.06	-1.85	Gbp8	NM_029509	-1.78	-1.56
Id2	NM_010496	-1.88	-1.76	Angptl1	ENSMUST00000027885	-3.39	-1.55
Plcl1	NM_001114663	-1.69	-1.71	Scn7a	NM_009135	-2.48	-1.55
Maoa	NM_173740	-1.68	-1.70	Olfr988	ENSMUST00000111597	-1.62	-1.54
Hey2	NM_013904	-5.09	-1.63	Vmn1r27	NM_134436	-1.67	-1.53
Adra1d	NM_013460	-3.44	-1.59	Rcan2	NM_030598	-3.09	-1.50
Soat2	ENSMUST00000023806	-1.81	-1.59				

Supplementary Table 3. Primer sequences for qPCR used in this study.

Name		Sequences (5' to 3')
mGAPDH	F	CCTCTGGAAAGCTGT GGCGT
	R	TTGGCAGGTTTCTCCAGGCG
mNrK	F	CAAGTGTTGGTGAGGAAGAG
	R	GT AGAAT AAGGCGGT GAT GA
mMMP3	F	CAGACTTGTCCCGTTTCCAT
	R	GGTGCTGACTGCATCAAAGA
mMMP8	F	CCTATTTCTCGTGGCTGCTC
	R	CCCACGGAGTGTGGTAGTAG
mMMP12	F	CCAAGCATCCCATCTGCTAT
	R	GGTCAAAGACAGCTGCATCA
mCCL6	F	CCAAGACTGCCATTTCATTC
	R	AAGCAATGACCTTGTTCCCA
mCCL8	F	ACGCTAGCCTTCACTCCAAAA
	R	TTCCAGCTTTGGCTGTCTCTT
mCCL11	F	TCCACAGCGCTTCTATTCCT
	R	GCAGTTCTTAGGCTCTGGGTT
mCCL19	F	GACCTTCCCAGCCCCAACT

	R	CGGAAGGCTTTCACGATGTT
mCXCL1	F	GCTCCCTTGGTTCAGAAAATTG
	R	TCACCAGACAGGTGCCATCA
mCXCL2	F	TCAAGAACATCCAGAGCTTGAG
	R	TTCAGGGTCAAGGCAAACTT
mCXCL3	F	AGGCCCCAGGC TT CAGAT AAT
	R	AAT GCAGGTCCTT CATCAT GGT
mCXCL5	F	GCCCTACGGTGGAAGTCATA
	R	GTGCATTCCGCTTAGCTTTC
mCXCL9	F	GT GAAGAAGC T GAT GAAAGAAT G
	R	AAGCCTATGT ATT AAAGGCTGCT
